# Rapid generation of dental pulp stem cell-derived mineralized extracellular matrix for quantitative osteoclast resorption assays

**DOI:** 10.3389/fbioe.2026.1802347

**Published:** 2026-04-22

**Authors:** Min Wu, Mingmei Chen, Yan Ma, Renren Chen, Dandan Cao, Yan Shen, Shunyuan Lu, Chunlin Shen, Hongxin Zhang, Zhugang Wang, Lingyun Tang

**Affiliations:** 1 State Key Laboratory of Medical Genomics, Research Center for Experimental Medicine, Rui-Jin Hospital Affiliated to Shanghai Jiao Tong University School of Medicine, Shanghai, China; 2 State Key Laboratory of Medical Genomics, National Research Center for Translational Medicine at Shanghai, Shanghai Institute of Hematology, Ruijin Hospital Affiliated to Shanghai Jiao Tong University School of Medicine, Shanghai, China; 3 Ruijin Hospital Lu Wan Branch, Shanghai Jiao Tong University School of Medicine, Shanghai, China

**Keywords:** biomaterials, dental pulp stem cells, extracellular matrix, osteoclast resorption assays, quantitative platform

## Abstract

Osteoclasts are large, multinucleated cells responsible for bone resorption, playing a central role in bone development, remodeling, and the pathogenesis of metabolic bone diseases through the degradation of inorganic hydroxyapatite and the organic matrix which is primarily composed of collagen. In the study of osteoclast biology, quantitative evaluation of bone resorption capacity is a key indicator of functional maturation. However, currently available assays largely rely on natural bone substrates, such as large animal teeth, or synthetic mineralized materials, both of which present limitations in accessibility, uniformity, and quantitative analysis. Here, we describe a novel resorption assay based on a rapidly mineralized extracellular matrix (ECM) derived from mouse dental pulp stem cells (DPSCs). Primary DPSCs isolated from adult C57BL/6 mouse incisors exhibited robust expansion within 5 days and generated a structurally uniform, well-mineralized ECM after 7 days of osteogenic induction. In addition, immortalization of primary DPSCs via SV40T lentiviral transduction produced stable cell lines with consistent and sustained mineralization capacity. Following decellularization, H&E, Von Kossa, and Masson’s trichrome staining showed that the decellularized ECM formed a continuous mineralized matrix containing collagen-rich organic components. Scanning electron microscopy and mass spectrometry further identified multiple ECM proteins associated with bone-like extracellular matrix. This mineralized ECM supported the differentiation of multiple osteoclast precursors—including mouse and rat bone marrow mononuclear cells as well as human THP-1 cells—into tartrate-resistant acid phosphatase (TRAP)-positive multinucleated osteoclasts with characteristic F-actin ring formation and measurable resorption activity. Moreover, the combination of Von Kossa staining enabled convenient and quantitative assessment of resorption areas across entire culture wells. The platform sensitively detected dose-dependent modulation of osteoclast activity by RANKL and IFN-γ, confirming its functional responsiveness. Collectively, the DPSC-derived mineralized ECM provides a natural, economical, rapid, and scalable platform for quantitative osteoclast resorption assays, with broad applicability in mechanistic studies and high-throughput drug screening.

## 1 Introduction

The extracellular matrix is a non-cellular three-dimensional network that provides both structural support and biochemical signals essential for tissue and organ function (Frantz et al., 2010). In contrast to soft tissues, bone-derived ECM constitutes the major proportion of the dry weight of bone and confers mechanical strength to the skeleton (Alford et al., 2015). Bone ECM is primarily composed of type I collagen fibers and carbonated hydroxyapatite (HA), along with a smaller portion of non-collagenous proteins, including γ-carboxyglutamate-containing proteins, proteoglycans, glycoproteins, and small integrin-binding ligand N-linked glycoproteins (Lin et al., 2020; Schlesinger et al., 2020). Beyond serving as a structural scaffold, the ECM regulates cell adhesion, migration, proliferation, and differentiation, functions as a reservoir for growth factors, participates in mineral nucleation, and undergoes continuous remodeling during bone formation and homeostasis (Mansour et al., 2017; Salhotra et al., 2020).

Bone development and homeostasis are maintained through the coordinated activities of osteoblasts and osteoclasts, which are responsible for bone formation and bone resorption, respectively. Osteoblasts originate from mesenchymal stem cells (MSCs) and generate bone matrix through the deposition and mineralization of collagen and hydroxyapatite crystals (Kim et al., 2020). In contrast, osteoclasts are derived from the monocyte-macrophage lineage and differentiate into large, multinucleated cells specialized for bone resorption (Florencio-Silva et al., 2015). Osteoclast differentiation and activation depend on several essential factors, most notably receptor activator of nuclear factor-κB ligand (RANKL) and macrophage colony-stimulating factor (M-CSF) (Udagawa et al., 2021). Mature osteoclasts possess a specialized cytoskeletal organization that enables the formation of a sealed resorptive microenvironment at the bone surface (Soysa and Alles, 2016). Within this compartment, an actin ring surrounds the ruffled border, allowing osteoclasts to acidify the interface and degrade both the mineral and organic components of the bone matrix (Teitelbaum, 2011).

Given the central role of osteoclasts in bone remodeling, cell-based resorption assays are essential tools for studying osteoclast differentiation and function. Commonly used readouts include tartrate-resistant acid phosphatase (TRAP) staining, actin ring formation and the quantification of resorption pits (Bernhardt et al., 2017). Currently, the materials used for resorption assays primarily consist of natural bone/dentin slices or artificial calcium phosphate cement (CPC) (Lutter et al., 2010; Xu et al., 2017). CPC does not fully recapitulate the complexity of the native bone matrix, whereas bone/dentin slices are typically obtained from large mammals, such as pigs or cattle, and are therefore costly and difficult to standardize (Fiorino et al., 2025). Moreover, accurate quantification of resorption activity on bone slices generally requires electron microscopy or toluidine blue staining, which limits throughput and accessibility (Rumpler et al., 2013; Vesprey and Yang, 2016). An alternative strategy is to generate mineralized ECM *in vitro* using cells with osteogenic differentiation capacity. Osteosarcoma cell lines, such as SaOS-2, and primary human osteoblasts have been reported to produce calcified ECM after 3–4 weeks of osteogenic induction (Lutter et al., 2010). However, the prolonged induction period increases experimental cost and time and frequently results in heterogeneity in both differentiation and mineralization, thereby complicating quantitative analysis in subsequent osteoclast resorption experiments. Therefore, the identification of an accessible cell source capable of rapidly producing a homogeneous mineralized ECM remains a critical unmet need for the development of standardized and quantitative osteoclast resorption assays.

Dental pulp stem cells (DPSCs) are neural crest-derived cells with multipotent differentiation capacity and exhibit robust osteogenic potential (Chamieh et al., 2016; Liu et al., 2015). DPSCs can be isolated from dental pulp using minimally invasive procedures, and protocols for the isolation and expansion of DPSCs from human dental pulp and mouse incisors are well established (Ellis et al., 2014; Gronthos et al., 2000). Owing to their high proliferative capacity, multilineage differentiation potential, and immunomodulatory properties, DPSCs have been widely used in tissue engineering and regenerative medicine, including nerve repair, pulp regeneration, bone regeneration, and craniofacial defect repair (Chamieh et al., 2016; Liang et al., 2021; Mead et al., 2017). In the present study, we developed a rapid and efficient method for generating a mineralized bone-like ECM based on mouse DPSCs within 7 days. This approach provides a convenient, time-saving, and cost-effective ECM platform that closely mimics key features of the natural bone matrix. We further demonstrate that this DPSC-derived ECM is suitable for osteoclast differentiation and quantitative bone resorption analysis, offering a versatile platform for functional studies and drug screening applications.

## 2 Materials and methods

### 2.1 Animals

Three-month-old C57BL/6 mice and one-month-old Sprague-Dawley rats were purchased from Shanghai Model Organism Company (Shanghai, China). All animal procedures were reviewed and approved by the Institutional Animal Care and Use Committee of Ruijin Hospital affiliated to Shanghai Jiao Tong University School of Medicine (approval number: RJ2023076; Date: 8 June 2023), and were conducted in accordance with institutional guidelines for the care and use of laboratory animals.

### 2.2 Primary culture of DPSCs

Primary DPSC culture was performed with minor modifications as previously described (Tang et al., 2024). Briefly, 3-month-old C57BL/6 mice were euthanized and immersed in 75% ethanol for 3 min. The mandibles were dissected using forceps and a scalpel, and surrounding soft tissues were carefully removed with gauze. The mandibular bones were then gently fractured to expose the incisor roots, and dental pulp tissues were carefully separated from the roots. The isolated dental pulps were cut into small explants approximately 1 mm^3^ and plated in culture dishes with MesenCult medium (StemCell, 05513) supplemented with penicillin/streptomycin (100 IU/mL). Cultures were maintained at 37 °C in a humidified incubator with 5% CO2. The explants were left undisturbed for the first 48 h to allow tissue attachment and cell outgrowth. The medium was replaced after 3 days of culture. When migrating cells formed large clonal cell clusters around the tissue explants, passaging was initiated. Cells were washed three times with pre-warmed PBS (Gibco, 18912014) and dissociated with Accutase (Thermo, A1110501) at 37 °C for 3 min. Digestion was terminated by the addition of twice the volume of complete medium, followed by centrifugation. The cells were then resuspended and replated at a split ratio of 1:3. DPSCs were expanded for up to five passages; however, reduced proliferative and differentiation capacities were observed with increasing passage number. Unless otherwise stated, DPSCs within passage three were used for subsequent experiments. Cell surface marker expression was analyzed by flow cytometry to confirm DPSC identity (negative markers: CD31, CD34, CD45; positive markers: CD29, CD44, Sca-1; Supplementary Table S1). Representative flow cytometry plots are shown in Supplementary Figure S1.

### 2.3 Construction of immortalized DPSC clones

Passage 1 DPSCs were transduced with a lentiviral vector encoding SV40 large T antigen (SV40T-GFP-Puro) at a multiplicity of infection (MOI) of 10 in the presence of 5 μg/mL polybrene (Gibco). The cells were incubated with viral particles for 24 h, after which the medium was replaced with fresh complete medium. At 72 h post-transduction, transduction efficiency was assessed by GFP fluorescence using an inverted fluorescence microscope (Nikon, Japan). Puromycin (1 μg/mL; Gibco) was then added for selection, and the cells were maintained under selective conditions for 7 days. Non-transduced cells were used as negative controls to verify selection efficiency. GFP-positive cells were subsequently subjected to single-cell cloning by limiting dilution in 96-well plates at a density of 0.5–1 cell per well. Single-cell-derived clones were expanded and maintained in MesenCult medium supplemented with 1 μg/mL puromycin for subsequent experiments.

### 2.4 Mineralized ECM formation and decellularization

Primary DPSCs or immortalized DPSC clones were seeded into 96-well plates at a density of 2 × 10^4^ cells per well and cultured in MesenCult medium. Upon reaching approximately 90% confluence, the medium was replaced with osteogenic induction medium consisting of α-MEM supplemented with 10% fetal bovine serum, 50 μg/mL ascorbic acid (Sigma, A5960), 10 mM β-glycerol phosphate (Sigma, G9422), 10 nM dexamethasone (Sigma, D4902), and penicillin/streptomycin. The induction medium was refreshed every 2 days. Mineralized ECM deposition became detectable from day 3 of induction, and a uniform ECM suitable for the resorption assay was obtained by day 7. At this time point, decellularization was performed using 0.5% Triton X-100 and 20 mM ammonium hydroxide for 5 min. The ECM was washed once with PBS and twice with ddH_2_O for 10 min each, air-dried, and sterilized under ultraviolet irradiation for 2 h. All procedures were performed under sterile conditions. Decellularized ECM plates were stored at 4 °C until further use.

### 2.5 Primary culture of BMMs

Primary bone marrow-derived mononuclear cells (BMMs) were isolated as previously described with minor modifications (Tang et al., 2021). Briefly, mice were euthanized and immersed in 75% ethanol for 3 min. Femurs and tibias were collected, and the surrounding soft tissues were removed with sterile gauze. Bone marrow cells were flushed from the marrow cavities using PBS containing 2% fetal bovine serum and penicillin/streptomycin. After centrifugation at 300 *g* for 10 min, the cell pellet was resuspended in red blood cell lysis buffer (eBioscience, 00-4300-54) and incubated at room temperature for 5 min. The cells were then diluted with PBS, filtered through a 70 μm cell strainer and centrifuged again. The resulting pellet was resuspended in α-MEM containing 10% fetal bovine serum and penicillin/streptomycin and plated in 10-cm culture dishes. After 24 h, non-adherent cells were collected for subsequent osteoclast differentiation assays.

### 2.6 Osteoclast differentiation

BMMs were collected, centrifuged at 300 *g* for 5 min, resuspended, and counted using trypan blue exclusion. The cells were seeded in α-MEM supplemented with 10% fetal bovine serum, 30 ng/mL M-CSF (R&D, 416-ML-010), and penicillin/streptomycin at a density of 0.5–1 × 10^6^ cells/cm^2^ and cultured for 24 h to allow adherence. Osteoclast differentiation was then induced by replacing the medium with osteoclast induction (OCI) medium containing α-MEM, 10% fetal bovine serum, 30 ng/mL M-CSF, 100 ng/mL RANKL (R&D, 462-TR-010), and penicillin/streptomycin. The OCI medium was replaced every 2 days. THP-1 cells were cultured in RPMI-1640 medium (HyClone, SH30027) supplemented with 10% fetal bovine serum and seeded in a 96-well plate at a density of 5 × 10^4^ cells/well. Adherent macrophage-like cells were induced by treatment with 100 ng/mL phorbol-12 myristate-13 acetate (PMA, Sigma, 524400) for 48 h (Li et al., 2017), followed by culture in OCI medium with medium changes every 2 days.

### 2.7 TRAP and actin ring staining

The cells were fixed with 4% paraformaldehyde solution for 15 min at room temperature and washed with PBS. TRAP staining was performed using an Acid Phosphatase Activity Detection Kit (Sigma, 387A) according to the manufacturer’s instructions. For actin ring staining, cells were permeabilized with 0.1% Triton X-100 in PBS for 15 min, washed three times with PBS, and stained with rhodamine-phalloidin (Invitrogen, R451) and DAPI (Sigma, D9542) for 30 min. Fluorescence images were acquired using multifunctional imaging detector BioTek Cytation5 (Agilent, United States).

### 2.8 Histological staining

Mineralized ECM was fixed with 4% paraformaldehyde for 15 min at room temperature and washed with deionized water. Samples were incubated with 5% silver nitrate solution for 30 min, washed, and then treated with 1% pyrogallol (Sangon, A606880) solution for 2 min. After additional washes with deionized water, plates were air-dried and imaged using BioTek Cytation5 (Agilent, United States). For H&E and Masson’s trichrome staining, decellularized ECM samples were fixed, embedded, sectioned, and stained according to standard histological procedures.

### 2.9 Scanning electron microscope

Decellularized ECM samples, with or without osteoclast or BMM culture, were fixed with 5% glutaraldehyde at 4 °C overnight. The samples were rinsed three times with 0.1 M phosphate buffer (pH 7.4) for 15 min each, post-fixed with 1% osmium tetroxide in the same buffer for 1–2 h at room temperature in the dark, and then rinsed three times again. Samples were dehydrated through a graded ethanol series (30%, 50%, 70%, 80%, 90%, 95%, 100%, and 100%) for 15 min at each step, followed by treatment with isoamyl acetate for 15 min. After critical point drying (Quorum K850), the samples were mounted on conductive carbon tape, sputter-coated with gold using an ion sputter coater (Hitachi MC1000), and examined using a scanning electron microscope (Hitachi SU8100) at 3.0 kV.

### 2.10 Proteomic analysis of decellularized ECM

Decellularized ECM samples were dissolved in 8 M urea and analyzed by LC–MS/MS using three biological replicates. Raw data were searched against the UniProtKB *Mus musculus* database using PEAKS Online 12. Protein abundance data were summarized at the gene level, and the mean abundance across three biological replicates was calculated for each entry. ECM-associated candidate proteins were ranked according to mean abundance, and the top proteins were selected for downstream visualization (Supplementary Table S2). Heatmaps and bar plots were generated using the online platform Bioinformatics (https://www.bioinformatics.com.cn; last accessed on 10 December 2024). For the heatmap, abundance values were log10-transformed and row-normalized as Z-scores.

### 2.11 Measurement and statistical analysis of mineralized ECM

Quantification of osteoclast-mediated resorption on mineralized ECM was performed using ImageJ software. Images were converted to grayscale, and pixels with grayscale values ranging from 0 to 50 were defined as mineralized areas for area measurement. All experiments were performed with at least four independent replicates. Statistical analysis was performed using unpaired two-tailed Student’s t-test, and *p* < 0.05 was considered statistically significant. Data analysis was conducted using GraphPad Prism version 9.1.1 (GraphPad Software, La Jolla, CA, United States).

## 3 Results

### 3.1 Mouse DPSCs are easily obtainable and can be rapidly expanded

**FIGURE 1:**
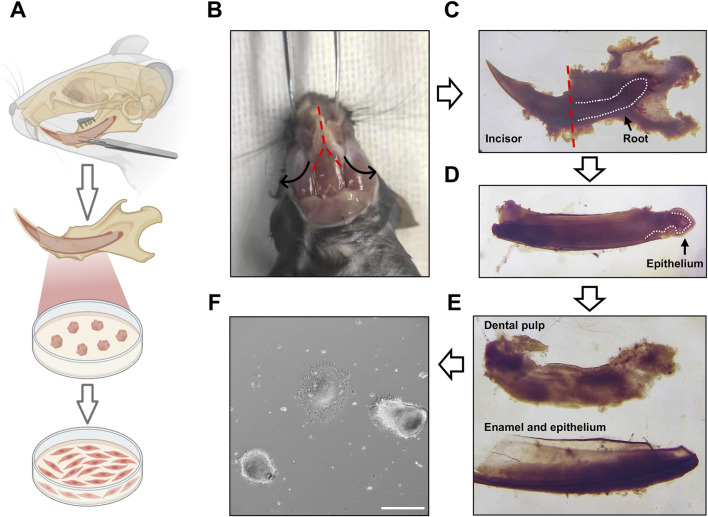
Isolation and expansion of DPSCs from mouse incisors. **(A)** Schematic illustration of the explant-based isolation procedure for adult C57BL/6 mouse incisors. **(B)** The lower mandible was isolated using forceps and a scalpel. The red dashed line indicates the incision site for the scalpel. The black arrows indicate the direction of removal of adjacent tissues. **(C)** The incisor root was isolated by breaking the mandible bone using a scalpel. The white dashed line indicates the tooth germ. The red dashed line indicates the position of the incisal cut, adjacent to the first molar. **(D)** Schematic diagram of separated incisor roots. The white dashed line indicates the boundary between the epithelium and mesenchymal compartments in the cervical loop. **(E)** The epithelial tissue was carefully removed, and the dental pulp tissue was separated from the root. The dental pulp tissue was cut into 0.5 mm^3^ explants and cultured in medium at 37 °C with 5% CO_2_. **(F)** After 24 h of incubation, the cells migrated outward from the tissue explants. Scale bar: 300 µm.

**FIGURE 2:**
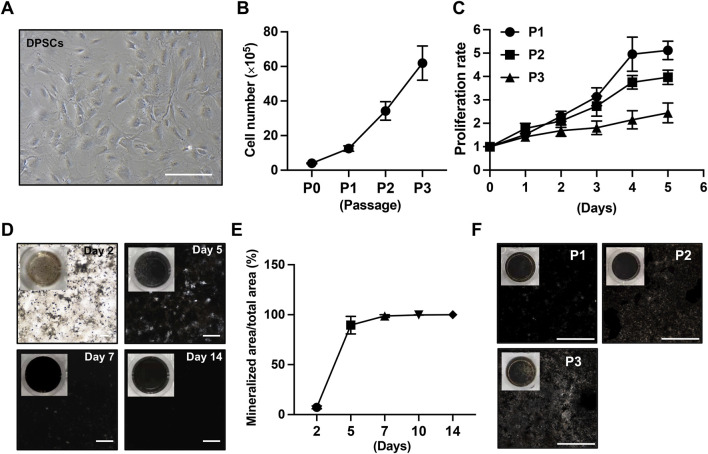
Expansion and mineralization capacity of primary mouse DPSCs. **(A)** Representative phase-contrast image showing the fibroblast-like morphology of primary DPSCs at passage 1. Scale bar: 200 µm. **(B)** Cell counts of the first three passages of DPSCs obtained from the primary culture of the lower incisors of a 3-month-old C57BL/6 mouse (n = 6). **(C)** The proliferative capacity of the first three passages of DPSCs was evaluated using the CCK-8 assay (n = 6). **(D)** Time-course analysis of ECM mineralization of passage 1 DPSCs assessed by Von Kossa staining at the indicated days following osteogenic induction. The overall view of the culture well is in the top-left corner of each image. Scale bar: 1,000 µm. **(E)** Quantification of the mineralized area shown in **(D)** (n = 3). **(F)** Comparison of ECM mineralization capacity of DPSCs at passages 1–3 after 7 days of osteogenic induction. Scale bar: 1,000 µm.

Mouse incisors provide an accessible and reliable source of dental pulp stem cells. In this study, DPSCs were isolated from the incisors of 3-month-old C57BL/6 mice. Although DPSCs derived from younger mice (3 weeks old) are generally easier to obtain and exhibit higher proliferative capacity, preliminary observations indicated that their mineralization potential was lower than that of DPSCs isolated from adult mice (3 months old). Therefore, adult mice were selected as donors for subsequent experiments. DPSCs were isolated using the explant culture method described in the Materials and Methods section (Figures 1A–F). Rather than being enzymatically dissociated into single cells, dental pulp tissue was cut into small explants (∼1 mm^3^) and cultured directly. This approach yielded a higher number of DPSCs with enhanced proliferative capacity, likely due to reduced cellular stress associated with enzymatic digestion. Using this protocol, a single 3-month-old C57BL/6 mouse yielded approximately 4 × 10^5^ primary DPSCs at passage 0. These cells displayed a flattened, fibroblast-like morphology (Figure 2A). After three passages, approximately 6 × 10^6^ DPSCs were obtained within 2 weeks (Figure 2B). Flow cytometric analysis confirmed that the isolated cells were negative for endothelial and hematopoietic markers (CD31, CD34 and CD45) and positive for mesenchymal stem cell markers (CD29, CD44, Sca-1), with more than 94% of cells expressing the positive markers (Supplementary Figures S1A,B). Similar to other mesenchymal stem populations, the proliferative capacity of DPSCs gradually decreased with increasing passage numbers, with a noticeable reduction observed at passage 3 (Figure 2C). These results indicate that DPSCs isolated from adult mouse incisors can be rapidly expanded *in vitro* while maintaining mesenchymal stem cell characteristics during early passages.

### 3.2 DPSCs rapidly form a homogeneous mineralized extracellular matrix

To evaluate the mineralized ECM-forming capacity of DPSCs, passage 1 DPSCs were seeded into 96-well plates at a density of 2 × 10^4^ cells/well and subjected to osteogenic induction upon reaching 80%–90% confluence. Mineral deposition was assessed at multiple time points using Von Kossa staining. Mineralized ECM became detectable as early as day 2 following induction. By day 5, approximately 80% of the well area was mineralized, and near-complete mineralization was observed by day 7 (Figures 2D,E). Extending the induction period to 14 days did not substantially increase the mineralized area (Figures 2D,E). The mineralization capacity of DPSCs at different passages was further examined. DPSCs at passages 1–3 were all capable of forming well-developed mineralized ECM by day 7 of induction, although a gradual decline in mineralization efficiency was observed with increasing passage number (Figure 2F). Nevertheless, DPSCs within the first three passages consistently generated sufficiently mineralized ECM suitable for downstream resorption assays. Compared with previously reported approaches using osteoblast cell lines to generate mineralized ECM over 4 weeks (Lutter et al., 2010), DPSCs exhibited a markedly accelerated mineralization profile. Following complete mineralization, cellular components were removed by decellularization, yielding an acellular ECM suitable for osteoclast resorption experiments. Collectively, these results demonstrate that DPSCs derived from adult mice rapidly produce a homogeneous mineralized ECM *in vitro*.

### 3.3 Immortalized DPSCs retain the capacity for mineralized extracellular matrix formation

**FIGURE 3:**
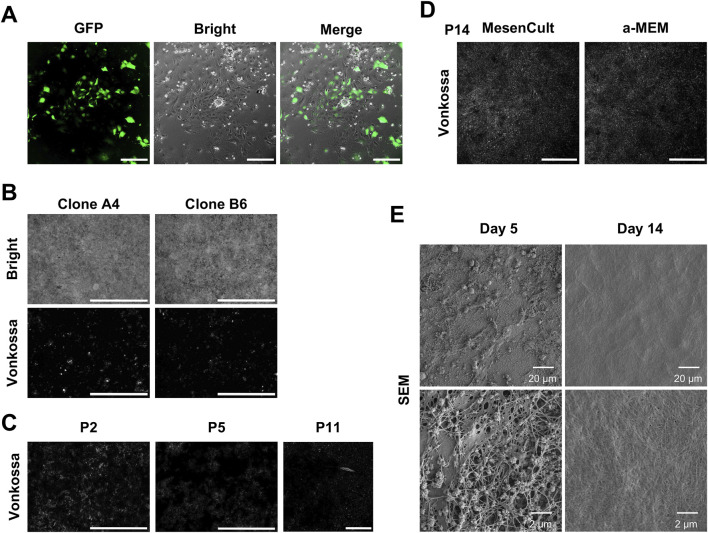
Establishment of immortalized DPSC lines and characterization of mineralized ECM. **(A)** Passage 1 DPSCs were examined under a fluorescence microscope for GFP-positive cells 72 h after SV40T lentivirus infection. Scale bar: 300 µm. **(B)** Two monoclonally derived immortalized DPSC clones capable of forming a well-mineralized extracellular matrix. Scale bar: 1,000 µm. **(C)** Clone A4 maintained a robust capacity for mineralized extracellular matrix formation after 11 passages. Scale bar: 1,000 µm. **(D)** Comparison of ECM mineralization by clone A4 cultured in MesenCult and α-MEM medium. Scale bar: 1,000 µm. **(E)** Scanning electron microscopy images showing the ultrastructural features of decellularized DPSC-derived ECM at the indicated induction time points.

Although primary DPSCs exhibited robust mineralization capacity, both proliferative and differentiation potential declined with increasing passage number. To overcome this limitation, immortalized DPSC cell lines were generated via SV40 large T antigen lentiviral transduction followed by puromycin selection (Figure 3A). Monoclonal populations were established using limiting dilution, resulting in 12 independent immortalized DPSC clones derived from single cells (Figure 3B; Supplementary Figure S2A). Among these clones, two (designated A4 and B6) consistently formed uniform mineralized ECM within 7 days of osteogenic induction (Figure 3B). To assess the long-term stability of mineralization capacity, clone A4 was subjected to serial passaging. This clone maintained robust ECM mineralization ability through at least 11 passages (Figure 3C; Supplementary Figure S2B). In addition, to reduce culture costs, MesenCult medium was replaced with α-MEM supplemented with 10% FBS. No apparent differences in mineralization capacity were observed between the two culture conditions after 14 passages (Figure 3D). To further clarify that immortalized DPSCs can consistently produce homogeneous ECM, we assessed the homogeneity within multi-well plates and between different batches. The results showed that ECM prepared from the same batch exhibited good homogeneity across multi-well plates, and consistent homogeneity was also maintained among ECMs derived from DPSCs with different passage numbers and different immortalized clones (Supplementary Figures S4A–D). These findings indicate that this method possesses the stability required for quantitative detection and the scalability for high-throughput applications.

### 3.4 Histological and compositional characterization of the DPSC-derived ECM

**FIGURE 4:**
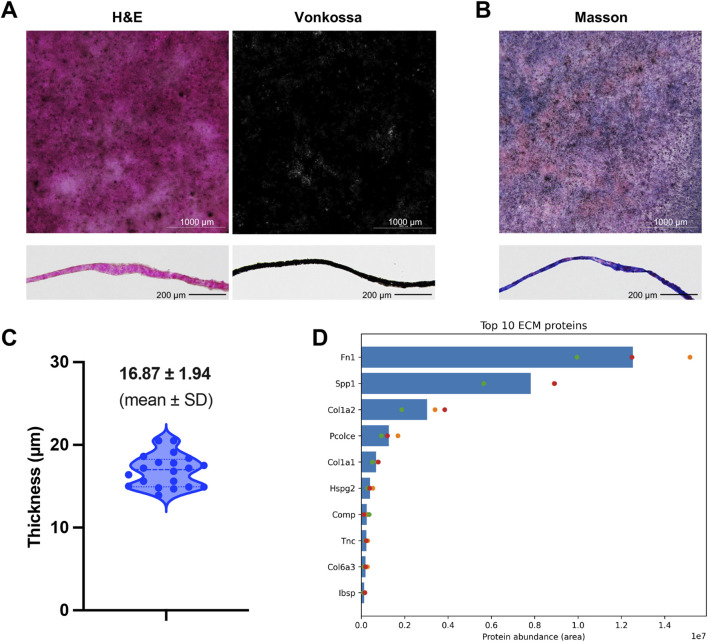
Histological and proteomic characterization of decellularized DPSC-derived ECM. **(A)** Representative images of H&E staining and Von Kossa staining of decellularized DPSC-derived ECM in 96-well plates (upper panels) and corresponding transverse sections (lower panels). H&E staining shows the overall distribution and continuity of the ECM, while Von Kossa staining indicates mineral deposition within the matrix. **(B)** Representative images of Masson’s trichrome staining of decellularized DPSC-derived ECM in whole-well view (upper panel) and transverse section (lower panel), demonstrating the presence of collagen-rich organic matrix. **(C)** Quantification of ECM thickness measured on transverse sections. Data are presented as individual measurements with a violin plot distribution; the mean thickness was 16.87 ± 1.94 μm (mean ± SD). **(D)** Proteomic analysis of the top 10 most abundant ECM-associated proteins identified in decellularized DPSC-derived ECM. Bars indicate mean protein abundance, and dots represent individual biological replicates. Scale bars: 1,000 μm (upper panels in **A, B**) and 200 μm (lower panels in **A, B**).

To further clarify that the ECM produced by DPSCs exhibited a bone-like morphology, we conducted histological and mass spectrometry analyses. First, H&E staining revealed a continuous, cell-free eosinophilic layer with a relatively uniform distribution across the culture surface (Figure 4A). Consistently, Masson’s trichrome staining demonstrated that the ECM contained abundant collagen-rich organic matrix (Figure 4B). Cross-sectional observation further showed that the matrix formed a compact lamellar-like layer on the plate surface rather than scattered amorphous deposits (Figures 4A,B; Supplementary Figure S3A). Quantitative measurement of ECM thickness showed a mean thickness of 16.87 ± 1.94 μm (n = 20) (Figure 4C), supporting the reproducibility and structural uniformity of the mineralized matrix. In addition, decellularized ECM was examined by scanning electron microscopy. The results revealed that on day 5 of differentiation, the ECM formed by DPSCs displayed a fibrous mineralizing morphology reminiscent of dentin/bone-like matrix (Tam and Pilliar, 1994) (Figure 3E). By day 14 of differentiation, a highly uniform ECM had formed (Figure 3E). To further define the composition of the decellularized DPSC-derived matrix, we performed mass spectrometry analysis on three biological replicates. The matrix contained abundant ECM-associated proteins, including FN1, SPP1, COL1A1, COL1A2, HSPG2, COL6A3, IBSP, COMP, TNC, and PCOLCE (Figure 4D; Supplementary Figure S3B; Supplementary Table S2). These findings support that the DPSC-derived ECM contains both structural and mineralization-related organic matrix components.

### 3.5 DPSC-derived ECM is suitable for osteoclast differentiation

**FIGURE 5:**
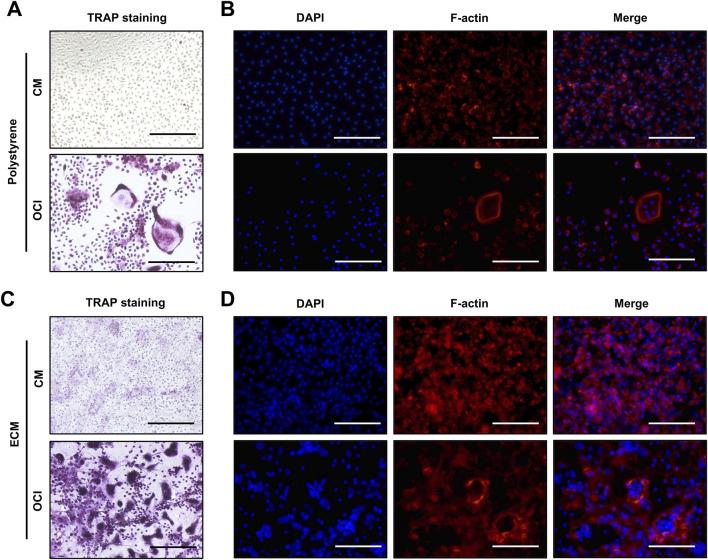
Osteoclast differentiation on DPSC-derived mineralized ECM. **(A)** Mouse BMMs were cultured on polystyrene in BMM culture medium or OCI medium for 7 days and analyzed by TRAP staining. **(B)** Mouse BMMs were cultured on polystyrene in BMM culture medium or OCI medium for 7 days and analyzed by F-actin staining. **(C)** Mouse BMMs were cultured on DPSC-derived ECM in culture medium and OCI medium for 7 days and analyzed by TRAP staining. **(D)** Mouse BMMs were cultured on DPSC-derived ECM in culture medium and OCI medium for 7 days and analyzed by F-actin staining. Mature osteoclasts showed TRAP positivity, multinucleation, and characteristic F-actin rings. F-actin was visualized by rhodamine-phalloidin (red), and nuclei were stained with DAPI (blue). CM, BMM culture medium; OCI, osteoclast induction medium. Scale bars: 500 µm.

To assess whether the DPSC-derived ECM is suitable for osteoclast differentiation, primary mouse bone marrow mononuclear cells (BMMs) were cultured on decellularized ECM or polystyrene surfaces under identical conditions. Cells were maintained either in BMM culture medium or osteoclast induction medium for 7 days. TRAP staining revealed that, in the presence of OCI medium, BMMs differentiated into multinucleated, TRAP-positive osteoclasts on both polystyrene and DPSC-derived ECM surfaces (Figures 5A,C). Notably, osteoclasts cultured in the ECM appeared more abundant and displayed morphological features more closely resembling those observed on natural bone substrates. Consistent with these observations, phalloidin staining demonstrated the formation of characteristic F-actin rings in osteoclasts differentiated on the ECM (Figures 5B,D), indicating the establishment of functional resorptive structures. In contrast, cells maintained in BMM culture medium did not exhibit TRAP positivity or actin ring formation on either surface (Figures 5A–D). These results demonstrate that DPSC-derived ECM effectively supports osteoclast differentiation and maturation.

### 3.6 Bone resorption by osteoclasts derived from multiple precursor sources

**FIGURE 6:**
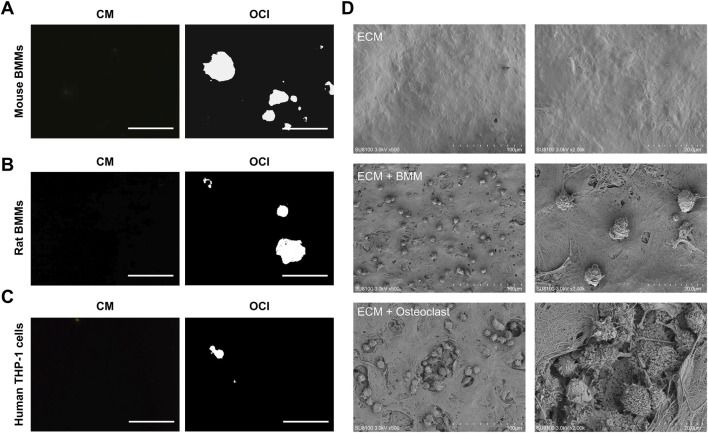
DPSC-derived ECM is compatible with osteoclast-mediated bone resorption from different precursor sources. **(A–C)** Mouse BMMs **(A)**, rat BMMs **(B)**, and human THP-1 cells **(C)** were plated on DPSC-derived ECM and cultured in BMM culture medium or OCI medium for 14 days. The white area indicates the region of bone resorption caused by osteoclasts. Calcified ECM was visualized by Von Kossa staining. Scale bars: 500 µm. **(D)** Scanning electron microscopy revealed ECM, BMMs and osteoclasts on DPSC-derived ECM.

To evaluate the applicability of the DPSC-derived ECM across different osteoclast precursor populations, primary mouse BMMs, primary rat BMMs, and human THP-1 cells were cultured on the ECM under osteoclast induction conditions for 14 days. Von Kossa staining showed distinct resorption areas in the mineralized ECM for all three cell types (Figures 6A–C). Mouse BMMs and rat BMMs exhibited robust resorptive activity, whereas THP-1 cell-derived osteoclasts displayed comparatively weaker resorption. This difference is consistent with the limited osteoclast differentiation efficiency reported for THP-1 cells. Further examination by scanning electron microscopy revealed that mature osteoclasts were able to degrade and resorb DPSC-derived ECM, forming bone resorption pits (Figure 6D). In contrast, cells maintained in BMM culture medium did not exhibit resorptive activity (Figures 6A–D). These findings indicate that the DPSC-derived ECM is compatible with osteoclast differentiation and resorptive activity across multiple species and precursor cell types.

### 3.7 Quantitative analysis of osteoclast resorption on DPSC-derived ECM

**FIGURE 7:**
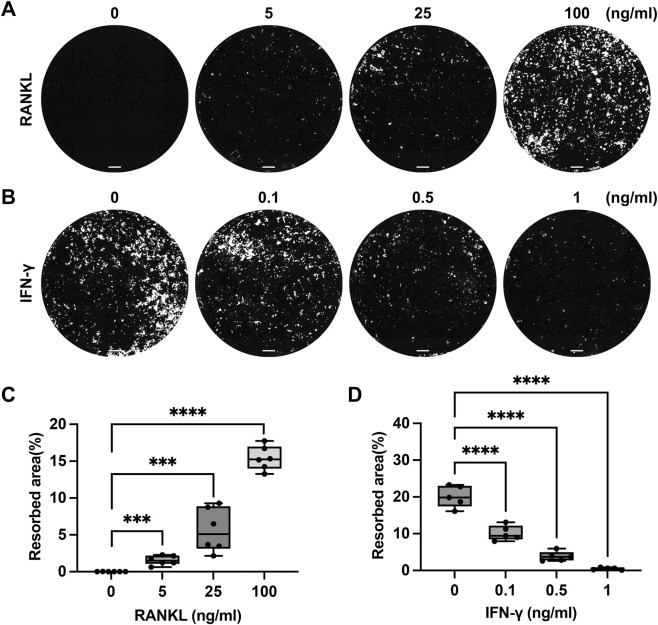
Quantitative assessment of osteoclast-mediated resorption on DPSC-derived ECM **(A)** Representative Von Kossa-stained images showing resorption areas of mouse osteoclasts following treatment with increasing concentrations of RANKL. **(B)** Representative Vonkossa-stained images showing resorption areas following treatment with increasing concentrations of IFN-γ. **(C)** Quantification of resorption area in response to RANKL treatment. **(D)** Quantification of resorption area in response to IFN-γ treatment. ***, p < 0.001, ****, p < 0.0001. Scale bars: 500 µm.

To assess the suitability of the DPSC-derived ECM for quantitative analysis of osteoclast function, the effects of cytokines known to modulate osteoclast differentiation were examined. RANKL is a cytokine that belongs to the tumor necrosis factor (TNF) family and plays a key role in osteoclast differentiation by activating signaling pathways that induce the expression of transcription factors essential for osteoclastogenesis (Wada et al., 2006). IFN-γ inhibits osteoclast differentiation directly by decreasing c-Fos and NFATc1 expression or by increasing TRAF6 degradation (Lai et al., 2022). It also indirectly inhibits osteoclast differentiation by stimulating osteoprotegerin (OPG) production, which competes with RANK for RANKL binding and inhibits osteoclastogenesis (Takayanagi et al., 2002). Mouse BMMs were cultured on the ECM in the presence of increasing concentrations of RANKL or IFN-γ. Von Kossa staining and subsequent quantification demonstrated a dose-dependent increase in resorption area with increasing RANKL concentrations (0, 5, 25, and 100 ng/mL) (Figures 7A,C). In contrast, IFN-γ treatment resulted in a progressive reduction in resorption area, decreasing from approximately 20%–2% with increasing IFN-γ concentrations (0, 0.1, 0.5, and 1 ng/mL) (Figures 7B,D). These results demonstrated that the DPSC-derived ECM platform sensitively detects both stimulatory and inhibitory modulation of osteoclast-mediated bone resorption, supporting its utility for functional and quantitative osteoclast assays (Amarasekara et al., 2018).

## 4 Discussion

To establish a biologically relevant, economical, and versatile *in vitro* platform for assessing osteoclast differentiation and resorptive function, we developed a rapid resorption assay based on a DPSC-derived mineralized extracellular matrix. Bone ECM consists of approximately 60% inorganic and 40% organic compounds, primarily hydroxyapatite and type I collagen, together with a variety of mineral-binding non-collagenous proteins (Mansour et al., 2017). Functional osteoclasts resorb bone by interacting with this composite matrix, underscoring the importance of substrate composition in vitro resorption assays. Currently used substrates, including artificial calcium phosphate cement (CPC), natural bone slices, and a small amount of osteoblast-derived ECM, each present inherent limitations. CPC differs from native bone in mineral composition and Ca/P ratio, parameters known to influence osteoclast activity (Wang et al., 2021). Dentin slices, although biologically relevant, are costly, difficult to standardize, and challenging to quantify due to their complex surface topography. Osteoblast-derived ECM requires prolonged culture periods, typically several weeks, which increases experimental variability and limits throughput.

Based on the high proliferative capacity and osteogenic potential of DPSCs (Tang et al., 2024), we established a rapid method for generating a mineralized ECM suitable for osteoclast resorption assays. In this system, DPSCs seeded in 96-well plates produced a mineralized ECM within 7 days of osteogenic induction. Additional histological staining and mass spectrometry further support that the matrix is not merely an amorphous mineral deposit, but a biologically derived mineralized ECM containing both mineral deposition and collagen-rich organic matrix. Therefore, it more closely represents a mineral–organic biological substrate than purely inorganic coatings. Subsequent resorption experiments demonstrated the presence of TRAP-positive multinucleated osteoclasts, well-defined F-actin rings, and clearly identifiable resorption pits on the ECM surface. Compared with previously reported ECM-based assays that require extended differentiation periods, the DPSC-derived ECM described here substantially reduces preparation time while providing a mineralized matrix that supports functional osteoclast maturation. This time efficiency, together with the compatibility of the ECM with standard multiwell plate formats, facilitates quantitative analysis and experimental scalability.

The applicability of the DPSC-derived ECM was further demonstrated using osteoclast precursors from multiple species, including mouse and rat bone marrow–derived mononuclear cells and human THP-1 cells. All three precursor types could differentiate into resorptive osteoclasts on the ECM, although THP-1–derived osteoclasts exhibited comparatively weaker resorption activity. This observation is consistent with the known limitations of THP-1 cells in osteoclast differentiation; however, species mismatch between the mouse-derived ECM and human precursors may also have contributed to the reduced resorptive activity. Thus, the present findings support the practical cross-species applicability of the platform, but not full biological equivalence across species. Importantly, the ECM supported osteoclast differentiation without impairing the formation of characteristic cytoskeletal structures, indicating that the matrix provides a permissive and biologically relevant substrate for osteoclast function across species.

A major advantage of this platform lies in its capacity for quantitative assessment of osteoclast-mediated bone resorption. Using Von Kossa staining in combination with inverted microscopy, resorption areas could be readily visualized and quantified across entire culture wells. This approach avoids the need for specialized imaging techniques, such as scanning electron microscopy, and enables efficient analysis of multiple samples in parallel. The functional responsiveness of the assay was validated by examining the effects of RANKL and interferon-γ (IFN-γ), two well-characterized modulators of osteoclast differentiation. The observed dose-dependent enhancement and inhibition of resorption activity, respectively, demonstrate that the DPSC-derived ECM platform sensitively reflects changes in osteoclast function induced by cytokine signaling. These features make the platform particularly suitable for comparative functional analyses and screening applications in a standardized multiwell format.

Despite these advantages, several limitations of the present system should be acknowledged. DPSCs are primary cells and, as such, exhibit a finite proliferative capacity and gradual functional decline with increasing passage number. To address this limitation, we established immortalized DPSC lines that retained stable and robust mineralization capacity over extended passaging, thereby improving the reproducibility and scalability of ECM production. In the present study, these immortalized cells were used only as an *in vitro* matrix-producing cell source, and the final assay substrate was an acellular decellularized ECM rather than a living immortalized cell product. Nevertheless, SV40T-mediated immortalization may still affect genomic stability or broader cellular phenotype, and therefore the current system should be considered suitable for *in vitro* functional applications only. Although histological and compositional analyses showed that DPSC-derived ECM exhibits bone-like features, the current physicochemical characterization remains incomplete, including calcium/phosphate quantification, crystallinity assessment, and mechanical testing. In addition, the platform does not fully recapitulate the complexity of native trabecular bone ECM. Moreover, we acknowledge that the present study did not include direct head-to-head benchmarking against dentin slices, bone slices, or hydroxyapatite-coated substrates. This remains an important next step for future validation. Accordingly, this platform is best suited for comparative functional analyses and screening applications rather than for modeling all aspects of *in vivo* bone remodeling.

In summary, we developed a DPSC-derived mineralized ECM platform that provides a rapid, economical, and biologically relevant substrate for *in vitro* osteoclast resorption assays. This system supports osteoclast differentiation from multiple precursor sources and enables sensitive, quantitative evaluation of resorptive function. Owing to its compatibility with standard multiwell formats and straightforward readout, the platform has broad potential applications in osteoclast biology research, mechanistic studies, and high-throughput drug screening.

## Data Availability

The original contributions presented in the study are included in the article/Supplementary Material, further inquiries can be directed to the corresponding authors.
