# Supplementary material for: Rapid generation of dental pulp stem cell-derived mineralized extracellular matrix for quantitative osteoclast resorption assays
Source: Front Bioeng Biotechnol. 2026 Apr 22;14:1802347. doi: 10.3389/fbioe.2026.1802347 (PMC13176466; doi:10.3389/fbioe.2026.1802347)
Supplement: Supplementary file 1 [file Supplementaryfile1.docx]

**Rapid Generation of Dental Pulp Stem Cell-Derived Mineralized Extracellular Matrix for Quantitative Osteoclast Resorption Assays**

**Supplemental Material**


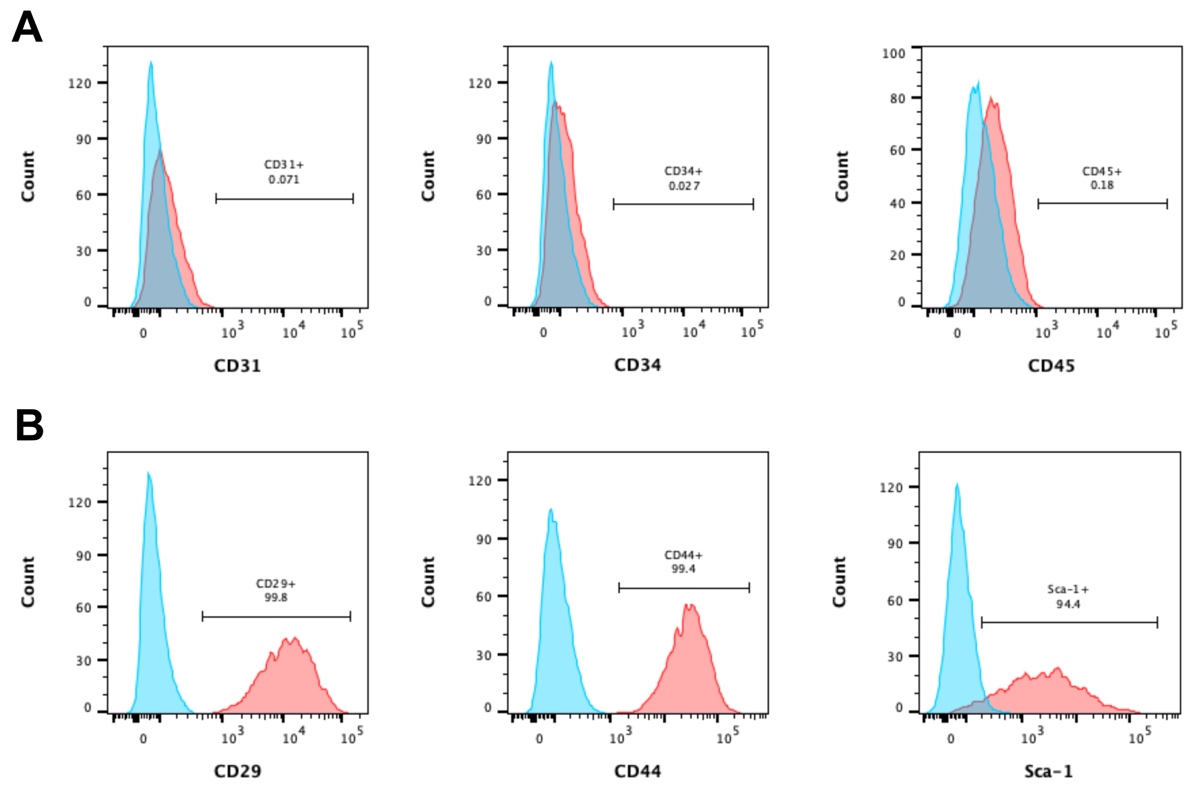


**Figure S1 Flow cytometric identification of mesenchymal stem cell characteristic surface markers in primary DPSCs.**

(A) Negative mesenchymal stem cell-associated surface markers (CD31, CD34 and CD45) in DPSCs. (B) Positive mesenchymal stem cell-associated surface markers (CD29, CD44 and Sca-1) in DPSCs.


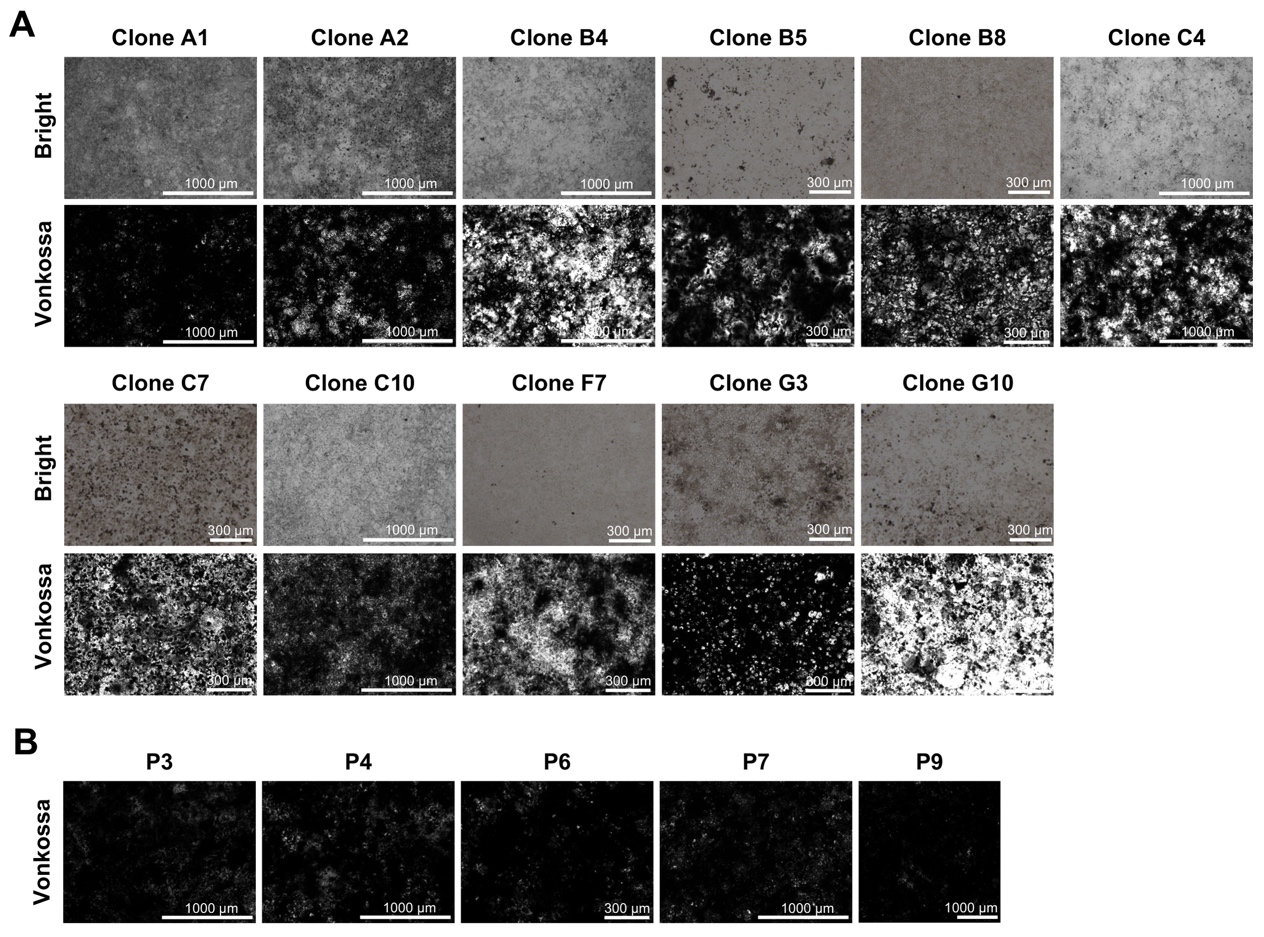


**Figure S2 Osteogenic differentiation capacity of immortalized DPSC clones.**

(A) After 7 days of osteogenic induction, immortalized DPSC monoclonal cells were imaged under bright-field microscopy (top) and after Von Kossa staining (bottom). (B) Clone A4 cells were subjected to osteogenic induction for 7 days at different passage numbers, and their mineralization capacity was assessed using Von Kossa staining.


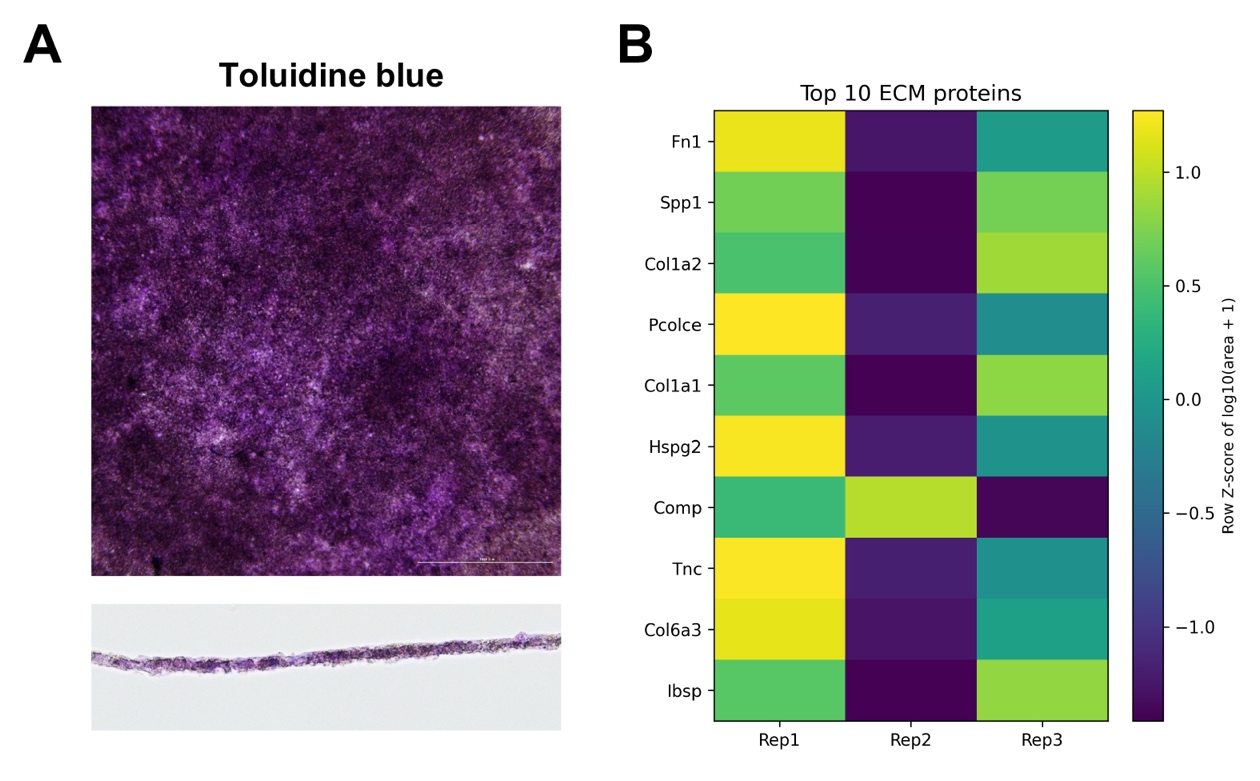


**Figure S3 ECM histochemical staining and mass spectrometry results**

(A) Toluidine blue staining of ECM in a 96-well plate (upper panel) and the corresponding cross-section of ECM (lower panel). (B) Heatmap showing the top 10 abundant ECM-associated proteins identified in decellularized DPSC-derived mineralized ECM by mass spectrometry. Protein abundance from three biological replicates was log10-transformed and row-normalized as Z-scores for visualization.


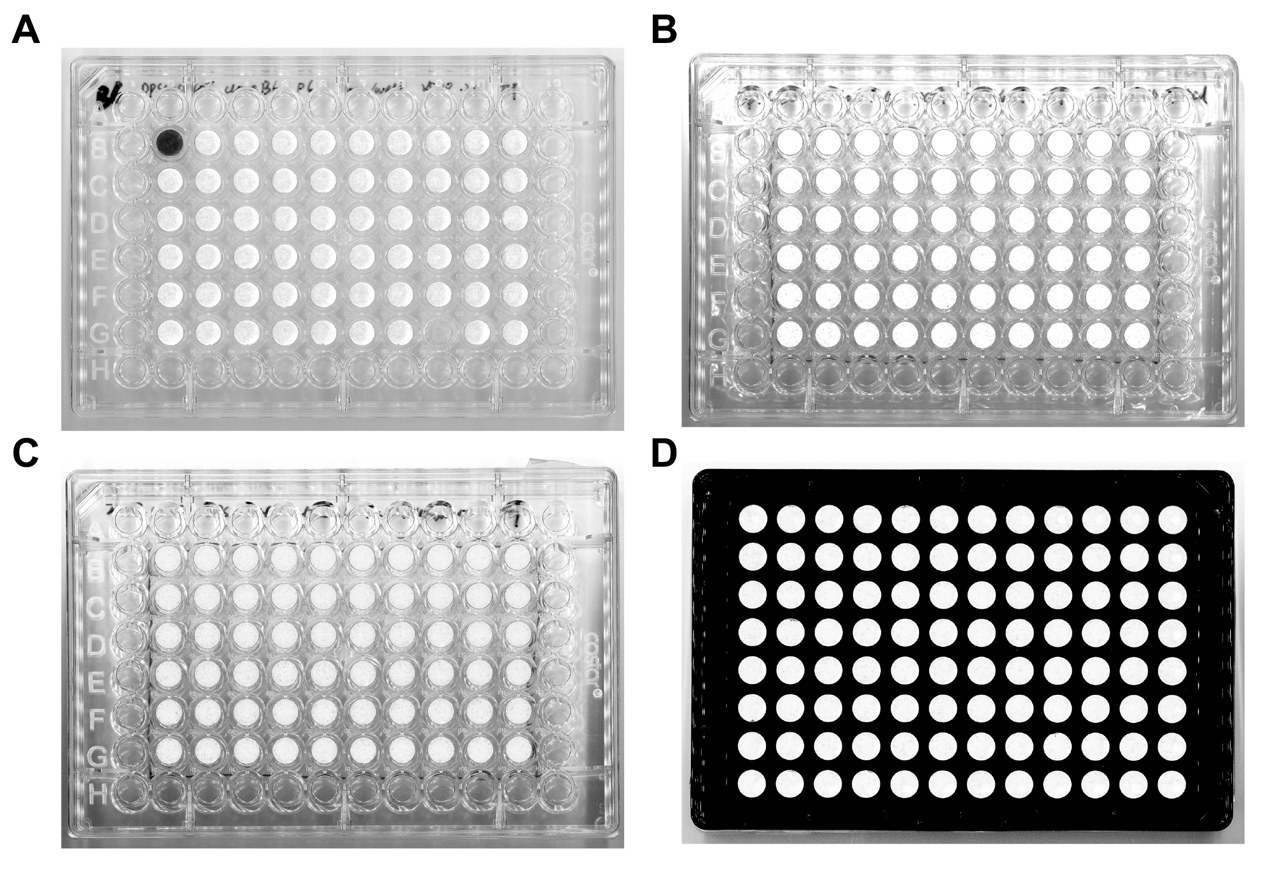


**Figure S4** High-throughput DPSC-derived ECM platform.

(A) The DPSC-derived ECM generated by clone B6 at passage 5 in a 96-well plate, with well B2 shown after Von Kossa staining. (B) DPSC-derived ECM generated by clone B6 at passage 9. (C) DPSC-derived ECM generated by clone A4 at passage 5 in a 96-well plate. (D) The DPSC-derived ECM generated by clone B6 at passage 9 in a 96-well plate adapter for an automated drug screening platform (Nunc, 165305).

**Table S1 Antibodies information**

|  | Name | Brand | Cat.NO. |
| --- | --- | --- | --- |
| **1** | **Anti-CD31-FITC** | eBioscience | 11-0311-82 |
| **2** | **Anti-CD34-FITC** | eBioscience | 11-0341-81 |
| **3** | **Anti-CD45-FITC** | eBioscience | 11-0451-82 |
| **4** | **Anti-CD29-PE** | eBioscience | 12-0291-81 |
| **5** | **Anti-CD44-PE/CY7** | BioLegend | 103030 |
| **6** | **Anti-Sca-1-APC** | eBioscience | 17-5981-82 |

**Table S2 Top 20 ECM proteins list**

| **Rank** | **Gene** | **n_detected** | **mean_area** | **cv_%** | **area_1** | **area_2** | **area_3** | **unique_**  **peptides** | **score** |
| --- | --- | --- | --- | --- | --- | --- | --- | --- | --- |
| 1 | *Fn1* | 3 | 12528275.7 | 20.8097733 | 15157627 | 9944000 | 12483200 | 156 | 297.66254 |
| 2 | *Spp1* | 3 | 7819163.33 | 24.0156223 | 8890900 | 5650890 | 8915700 | 60 | 242.01076 |
| 3 | *Col1a2* | 3 | 3038633.33 | 34.2294126 | 3402400 | 1865500 | 3848000 | 38 | 222.54202 |
| 4 | *Pcolce* | 3 | 1271833.33 | 30.3444477 | 1687000 | 924000 | 1204500 | 25 | 198.9715 |
| 5 | *Col1a1* | 3 | 682166.667 | 21.8263306 | 751100 | 511300 | 784100 | 31 | 213.26714 |
| 6 | *Hspg2* | 3 | 404330 | 30.201279 | 533830 | 291270 | 387890 | 124 | 283.9538 |
| 7 | *Comp* | 3 | 251200 | 49.0001039 | 276000 | 360000 | 117600 | 19 | 212.94652 |
| 8 | *Tnc* | 3 | 232420 | 22.5678177 | 287880 | 183610 | 225770 | 53 | 239.9448 |
| 9 | *Col6a3* | 3 | 197511.333 | 39.6889717 | 277880 | 121262 | 193392 | 59 | 244.88568 |
| 10 | *Ibsp* | 3 | 134333.333 | 30.1496492 | 152000 | 88000 | 163000 | 4 | 101.6611 |
| 11 | *Thbs2* | 3 | 125423.333 | 44.8361067 | 188300 | 108030 | 79940 | 14 | 229.55954 |
| 12 | *Col5a1* | 3 | 80400 | 66.4871674 | 138500 | 33300 | 69400 | 26 | 203.66034 |
| 13 | *Bgn* | 3 | 75343.3333 | 17.3767216 | 78410 | 60990 | 86630 | 3 | 196.31224 |
| 14 | *Fbln2* | 3 | 39866.6667 | 30.4146769 | 53200 | 36900 | 29500 | 25 | 200.4633 |
| 15 | *Sparc* | 3 | 33593.3333 | 16.4710798 | 31720 | 29240 | 39820 | 9 | 147.31776 |
| 16 | *Thbs1* | 3 | 29350 | 47.4699604 | 38040 | 36730 | 13280 | 1 | 254.82526 |
| 17 | *Dcn* | 3 | 11883.3333 | 105.2888 | 25762 | 1468 | 8420 | 5 | 122.57263 |
| 18 | *Col6a1* | 3 | 9541.66667 | 50.4467001 | 13530 | 4195 | 10900 | 6 | 132.95517 |
| 19 | *Fbln1* | 3 | 7700.33333 | 25.3607873 | 9954 | 6507 | 6640 | 3 | 90.96625 |
| 20 | *Mgp* | 3 | 5479 | 65.8742659 | 9510 | 4380 | 2547 | 3 | 94.407936 |
